# The prognostic significance of postoperative hyperbilirubinemia in cardiac surgery: systematic review and meta-analysis

**DOI:** 10.1186/s13019-022-01870-2

**Published:** 2022-05-26

**Authors:** Dev Raveendran, Jahan C. Penny-Dimri, Reny Segal, Julian A. Smith, Mark Plummer, Zhengyang Liu, Luke A. Perry

**Affiliations:** 1 Department of Anaesthesia and Pain Management, Royal Melbourne Hospital, 300 Grattan St, Parkville, VIC 3050 Australia; 2 Melbourne Medical School, University of Melbourne, Parkville, Australia; 3 Department of Surgery, School of Clinical Science, Monash University, Clayton, Australia; 4 Department of Surgery, Barwon Health, Geelong, Australia; 5 Department of Cardiothoracic Surgery, Monash Health, Clayton, Australia; 6 Centre for Integrated Critical Care, University of Melbourne, Parkville, Australia; 7 Intensive Care Unit, Royal Melbourne Hospital, Parkville, Australia

**Keywords:** Cardiopulmonary bypass, Hyperbilirubinemia, Jaundice, Length of stay, Prognostic biomarkers

## Abstract

**Background:**

Hyperbilirubinemia following cardiac surgery is a common phenomenon and is of emerging interest in prognostic factor research. This systematic review and meta-analysis evaluated the association between post-operative hyperbilirubinemia (PH) and mortality and morbidity in cardiac surgery patients.

**Methods:**

Ovid Medline and Ovid Embase were searched from inception to July 2020 for studies evaluating the prognostic significance of PH following cardiac surgery. Maximally adjusted odds ratios (OR) with associated confidence intervals were obtained from each study and pooled using random effects inverse variance modelling to assess in-hospital mortality. Standardised mean differences were pooled to assess Intensive Care Unit (ICU) and hospital length of stay (LOS). Qualitative analysis was performed to assess ventilation requirements and long-term mortality. Meta-regression was used to assess inter- and intra-study heterogeneity.

**Results:**

3251 studies satisfied the selection criteria, from which 12 studies incorporating 3876 participants were included. PH significantly predicted in-hospital mortality with a pooled OR of 7.29 (95% CI 3.53, 15.09). Multiple pre-defined covariates contributed to the prognostic significance of PH, however only aortic cross-clamp time (*p* < 0.0001) and number of transfusions (*p* = 0.0001) were significant effect modifiers. PH significantly predicted both ICU LOS (Mean difference 1.32 [95% CI 0.04–2.6]) and hospital LOS (Mean difference 1.79 [95% CI 0.36–3.21]). Qualitative analysis suggested PH is associated with increased post-operative ventilation requirements and reduced long-term survival rates.

**Conclusions:**

Hyperbilirubinemia is a cost-effective, widely available prognostic marker of adverse outcomes following cardiac surgery, albeit with residual sources of heterogeneity.

**Supplementary Information:**

The online version contains supplementary material available at 10.1186/s13019-022-01870-2.

## Introduction

Post-operative hyperbilirubinemia (PH), generally described as > 3 mg/dL, is a common complication following cardiac surgery. PH incidence varies between 10 and 40% depending on the severity of underlying cardiac disease and the type of surgery performed [1–4]. Moreover, PH has been associated with adverse patient outcomes such as prolonged ICU stay, new onset infection, low-output syndrome, and increased requirements for invasive ventilation and renal replacement therapy [5].

The aetiology of PH is debated and thought to be multifactorial. Cardiopulmonary bypass (CPB) is a recognised risk factor that can lead to hypoperfusion, systemic inflammation and haemolysis [6–8]. Additional risk factors include patient age, heart failure status, postoperative sepsis, and intra-operative administration of blood products [2, 9–11].

Despite advancements in CPB and anaesthesia techniques, the incidence of hyperbilirubinemia after cardiac surgery has not decreased since the first report in 1967 [9, 11, 12]. A recent study reported a 10% incidence with an associated mortality of 17.4% [5]. This mortality rate rises to 90% in cases when progression to hepatic failure is observed [1, 3, 13]. Moreover, the timing of bilirubin elevation post-surgery is of clinical importance with late-onset hyperbilirubinemia (> 7 days) being associated with increased mortality [5].

Given that plasma bilirubin assays are routinely performed after cardiac surgery and may be a predictor of adverse patient outcome we conducted a systematic review and meta-analysis to evaluate the prognostic value of PH following cardiac surgery.

## Methods

### Study design and registration

This systematic review and meta-analysis was constructed in accordance with the Preferred Reporting Items for Systematic reviews and Meta-Analysis (PRISMA) Statement [14], and conducted according to methodological guidance [15]. Details of the protocol of this prognostic research review were registered prospectively (PROSPERO ID CRD42020206068). There were no protocol deviations.

### Eligibility criteria

Eligible studies met the following criteria (a) randomized controlled trials, non-randomized controlled trials (case control or controlled cohort), observational studies (b) study population of adult patients (aged ≥ 18 years) (c) exposure to cardiopulmonary bypass for coronary artery bypass grafting (CABG), valvular surgery or combined CABG and valvular surgery (e) outcome measure of plasma bilirubin reported (f) outcome measure of mortality or morbidity measured. Studies involving organ transplant, ventricular assist devices, and extracorporeal membrane oxygenation were excluded.

### Search strategy

OVID Medline and OVID Embase were searched from inception to July 2020 using a set of a validated and comprehensive keywords and medical subject headings (MeSH) relating to ‘cardiac surgery,’ ‘hyperbilirubinemia’ and ‘mortality and morbidity’ (see Additional file 1). Reference lists from published articles were hand searched for potentially relevant studies. No restrictions were placed on language or publication year. The reference lists of the included studies were separately searched for further potential citations.

### Study selection

Two reviewers (DR and LP) independently screened titles and abstracts of all identified studies. Full text screening of potentially relevant studies was performed by the same reviewers with a third author (JPD) adjudicating any disagreements. The definition of hyperbilirubinemia was as defined by the authors in each study, if no definition was given a cut-off of 3 mg/dL (51.3 µmol/L) was used.

### Data extraction and management

Two reviewers (DR and LP) independently extracted the following information onto standardised forms: Study designs, population demographics, co-morbidities, operative details, proportion with PH, timing of PH peak, conjugated vs unconjugated phenotype of PH, ICU length of stay (LOS), hospital LOS, post-operative ventilation time and mortality following discharge **(**see Additional file 2). Where provided, maximally adjusted odds ratio (OR) for short term survival were used. Mean differences were used for continuous outcomes. Where studies stratified patients into more than two groups (e.g. tertiles or quartiles) we compared the upper most quantile against the cumulative lowermost quantiles.

### Assessment of methodological quality

The Prediction model Risk Of Bias Assessment Tool (PROBAST) was utilised to assess the methodological quality of the included studies. Assessment was performed by two review authors (DR and LAP) and disagreements were resolved through discussion with a third author (JPD). PROBAST is tailored for prognostic studies and assesses risk of bias across four domains: participants, predictors, outcome, and analysis [16, 17].

### Statistical analysis and data synthesis

We tabulated the maximally adjusted OR with associated 95% confidence intervals for each study assessing in-hospital mortality and generated a pooled OR using mixed-methods (generalised linear) inverse variance modelling. For continuous outcomes such as ICU and hospital LOS, we generated mean differences with 95% confidence intervals. Analysis was of post-operative ventilation times or long-term mortality was not performed due to the low number of reporting studies.

Chi-square statistics were used to estimate statistical heterogeneity for each outcome. Where there were greater than 10 studies reporting an outcome, we conducted a meta-regression to explore sources of statistical heterogeneity by inputting the following covariates: study year, age, proportion of males, bilirubin threshold (as defined by study authors), day of bilirubin measurement, cardiopulmonary bypass time, clamp time, number units of blood transfused and proportion with pre-operative liver disease. Where there were fewer than 10 studies reporting on an outcome, potential sources of clinical and statistical heterogeneity were explored qualitatively.

Publication bias was formally assessed by generating funnel plots. Visual testing of skew was performed, and funnel plot asymmetry was analysed using the Classical Egger test, fixed- and mixed-effects meta-regression models with p values [18, 19]. To further examine for suppression of non-significant studies, we constructed a contour enhanced funnel plot [20]. All analyses were performed using the R statistical package ‘metafor’, with figures generated using ‘ggplot2’ [21, 22].

## Results

### Search results

**Fig. 1:**
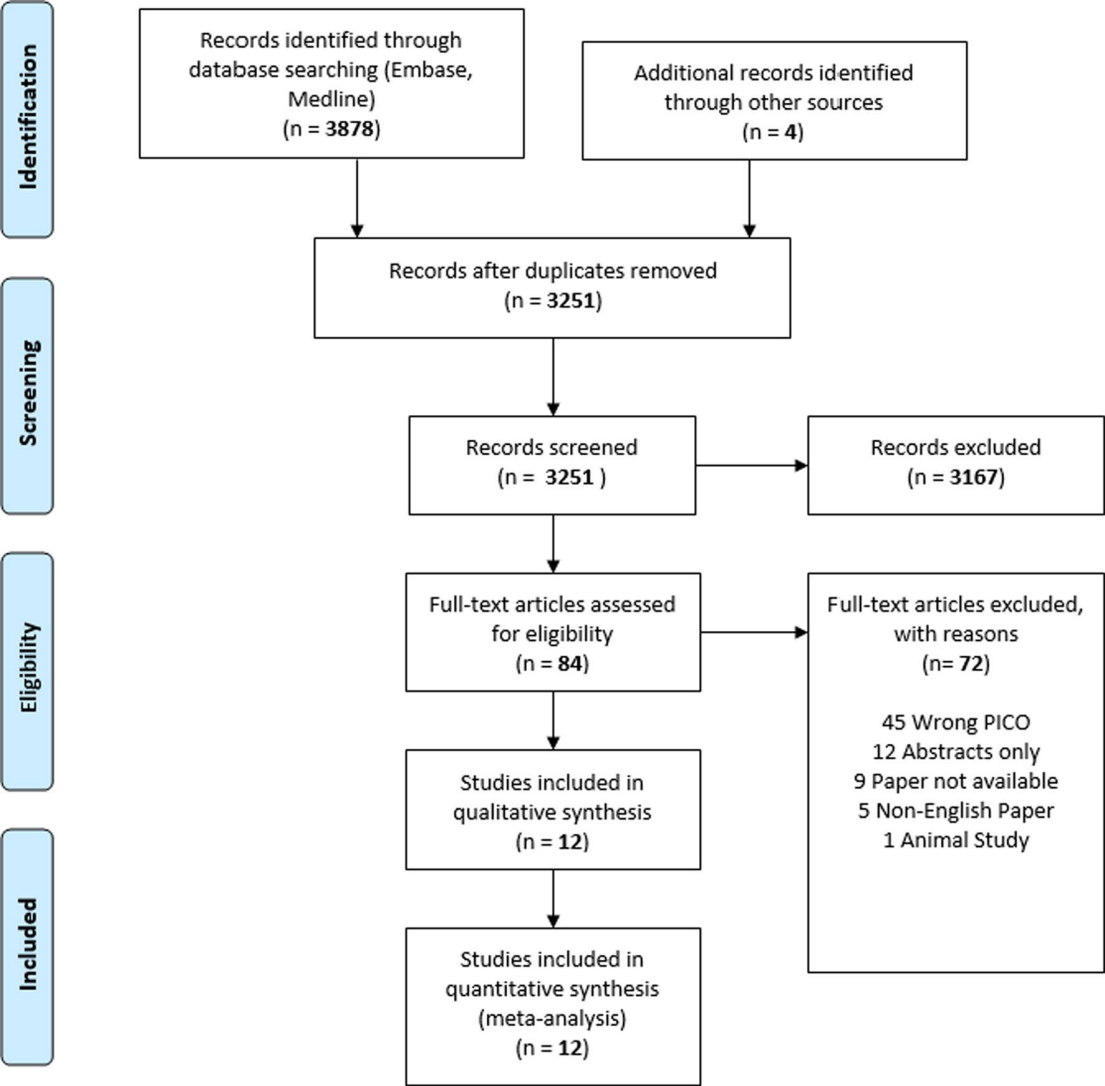
Prisma Flow chart

The search returned 3878 citations and an additional four relevant citations were found from other sources. The removal of duplicates resulted in 3251 unique studies. After title and abstract screening, 84 studies underwent full text review of which 12 studies were selected **(**see Fig. 1).

### Description of included studies

Study design, patient demographics, operative details, days of bilirubin measurement, presence of preoperative liver disease and number of units of blood transfused are detailed in (see Table 1). The 12 studies were published between 1983 and 2017 and included a total of 3876 participants [23–34]. All included studies reported post-operative bilirubin measurements. Four studies were retrospective [25, 27–29], and the other eight studies were prospective. The mean age ranged from 32 to 71 years with a high proportion of the participants being male. The threshold for hyperbilirubinemia ranged from 2 to 3 mg/dL. Seven studies set a threshold of 3 mg/dL [23–26, 30, 32, 33], four studies at 2 mg/dL [27, 29, 31, 34], and one study at 2.8 mg/dL [28]. Nine studies reported post-operative bilirubin levels for at least 7 days following surgery [23, 24, 26–28, 30, 31, 33, 34]. Two studies reported measurements up to 2 and 5 days respectively [25, 29], with one study not specifying the days of measurement [32]. Several of the pre-specified modifier covariates were inconsistently reported and are provided as an online supplement.

**Table 1:** Characteristics of Included Studies

Study ID	Study Design	Sample Size (N) Case mix	PH incidence % (n)	% Male and age (mean (SD))	Bilirubin threshold (mg/dL)	Minimum monitoring period (days)	Number of units of blood transfused in PH group (mean (SD))	Number of units of blood transfused in non-PH group (mean (SD))	CPB Time (mins)	Cross Clamp time (mins)	Outcomes reported
Collins [24]	Prospective (single centre)	N = 248	20% (50)	NR	3	7	10.1 (7.5)	5.3 (3.5)	91.96	NR	In-hospital mortality
		7% TV									
		60% MV									
		39% AV									
		38% CABG									
Wang [33]	Prospective (single centre)	N = 302	35% (106)	57% Male	3	7	NR	NR	121.2	70.3	In-hospital mortality
		30% CABG		51.87 (0.8)							ICU LOS
		32% Valve									
		24% Redo									
		6% CHD									
		7% Complex									
Chandra [34]	Prospective (single centre)	N = 77	26% (20)	70% Male	2	7	NR	NR	93.5	46.3	In-hospital mortality
		26% CABG		32.1% (16.4)							ICU LOS
		38% first time valve									Hospital LOS
		4% valve reoperation									
		27% congenital repair									
		50% reconstructive procedure									
Hosotsubo [27]	Retrospective (single centre)	N = 133	51% (68)	59% Male	2	14	6.85 (0.88)	4.0 (0.52)	NR	NR	In-hospital mortality
		38% CABG		60 (1.3)							ICU LOS
		48% valvular									
		14% Aneurysm									
An [23]	Prospective	N = 386	25% (96)	47% Male	3	7	3.56 (0.02)	2.15 (0.03)	116.8	70.6	In-hospital mortality
	(single centre)	57% valve cases		46.3 (1.14)							ICU LOS
		36% CHD cases									Ventilation Time
		4% combined cases									
		0% CABG									
Leacche [29]	Retrospective (single centre)	N = 136	NR	37% Male	2	5	NR	NR	154	99	In-hospital mortality
		40% CABG/valve		67(12)							
		29% CABG only									
		15% valve only									
		9% transplant									
Kraev [28]	Retrospective (single centre)	N = 826	9% (74)	71% Male	2.8	7	8.0 (10.0)	2.49 (2.77)	143	106	In-hospital mortality
		62% CABG		65 (13)							Hospital LOS
		6% Valve									Long-term Mortality
		32% other									
Vidal [32]	Prospective	N = 73	23% (16)	68% Male	3	NR	NR	NR	106	65	In-hospital mortality
	(single centre)	23% valve replacement		71 (11)							
		22% CABG									
		52% Combined									
		3% Other									
Nishi [30]	Prospective	N = 334	19% (63.46)	60% Male	3	7	NR	NR	183	NR	In-hospital mortality
	(single centre)	49% aortic valve		64.3 (14.9)							
		51% mitral valve									
		22% tricuspid									
		0% CABG									
Sharma [31]	Prospective	N = 476	25% (119)	NR	2	7	3.95 (0.68)	2.29 (0.67)	103.77	74.06	In-hospital mortality
	(single centre)	42% CABG									ICU LOS
		34% valve repair									Hospital LOS
		17% congenital									Ventilation Time
		6% Valve + CABG									
Diab [25]	Retrospective	N = 285	25% (71)	71% Male	3	2	NR	NR	135	NR	In-hospital mortality
	(single centre)	44% single aortic valve IE		62 (14.1)							Long-term mortality
		28% single mitral valve IE									
		27% both valves									
		21% prosthetic valve IE									
		0% CABG									
Golitaleb [26]	Prospective (single centre)	N = 600	25% (150)	55% Male	3	7	NR	NR	105	81.67	In-hospital mortality
		33% CABG		63.8 (8.94)							ICU LOS
		33% AVR + CABG									Hospital LOS
		33% MVR + CABG									

*AV* aortic valve, *AVR* aortic valve replacement, *CABG* coronary artery bypass grafts, *CHD* congenital heart disease, *IE* infective endocarditis, *MV* mitral valve, *MVR* mitral valve replacement, *NR* Not reported, *TV* tricuspid

Maximally adjusted OR for in-hospital mortality was reported in two studies [29, 32], unadjusted data were extracted from the remaining ten studies. We calculated standardised mean differences for ICU LOS from six studies [23–25, 27, 29, 30, 32, 33], and hospital LOS from four studies [26, 28, 31, 34].

### Methodological quality

**Fig. 2:**
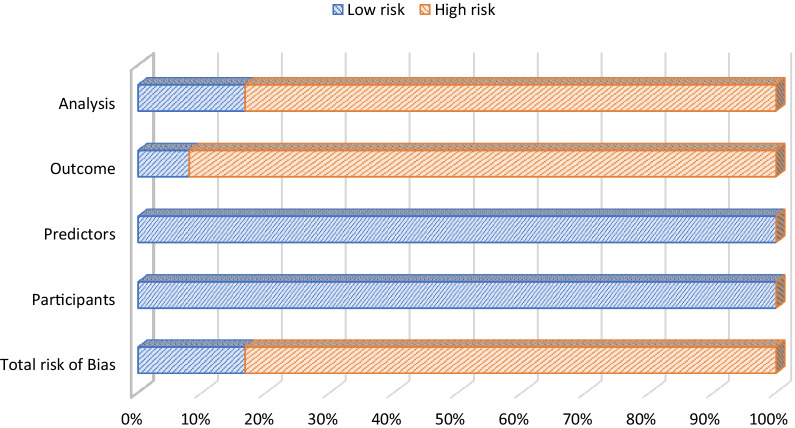
PROBAST risk of bias graph

The overall methodological quality of the studies was poor with only 2 studies having low risk of bias [28, 32], and 10 studies having high risk of bias (see Fig. 2). Studies reporting only unadjusted data such as frequency of deaths observed were deemed high risk due to the potential for unaccounted for significant confounding variables. The complete table of PROBAST scores for each included study is available in the (see Additional file 3).

### Meta-analysis

#### Quantitative analysis

##### In-hospital mortality

**Fig. 3:**
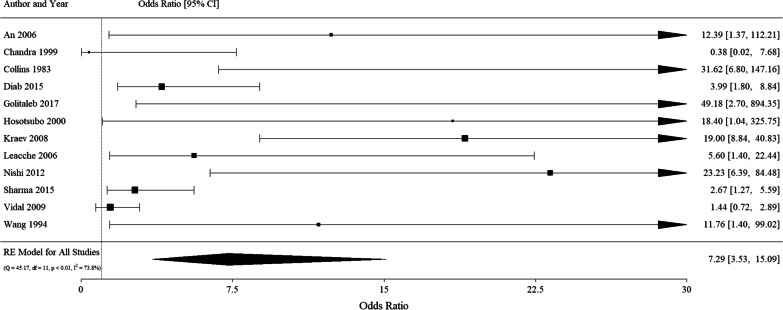
Forest plot for PH predicting in-hospital mortality. Arrowheads indicate continuing confidence intervals

All studies reported on hospital mortality. PH was strongly associated with in-hospital mortality following cardiac surgery (OR 7.29 [95% CI 3.53, 15.09]) **(**see Fig. 3). Between-study statistical heterogeneity was substantial (I^2^ 73.8%) with aortic cross clamp time and transfusion requirements being significant effect modifies on meta-regression analysis (see Table 2). The remaining pre-specified covariates were either not significant or not included in the meta-regression due to insufficient reporting **(**see Additional file 2). The variability introduced by the covariates and other study and patient-level factors partially contributes to the residual heterogeneity and the wide confidence interval. The mortality rates in the PH group was 13.08% (9.35) versus 2.21% (2.38) in the non-PH group (see Additional file 4).

**Table 2:** In-hospital mortality meta-regression results

Covariate	Study count	Co-efficient	95% CI	*p* value
Clamp time*	8	0.0556	0.0334–0.0778	< 0.0001
Transfusion Units*	8	0.6928	0.3393–1.0463	0.0001
Study year	12	− 0.037	− 0.1131 to 0.0390	0.3402
Age	10	0.0249	− 0.0647 to 0.1145	0.5864
Sex (Male)	10	− 2.7767	− 10.3351 to 4.7817	0.4715
Bilirubin threshold	12	1.0256	− 0.6223 to 2.6734	0.2225
Bilirubin monitoring period	7	0.1577	− 0.1212 to 0.4365	0.2678
CPB time	11	0.0159	− 0.0108 to 0.0427	0.2431
CABG proportion	12	− 0.2853	− 2.8252 to 2.2547	0.8258
Pre-operative liver disease	5	− 7.4876	− 16.3865 to 1.4113	0.0991

*CABG* Coronary artery bypass graft, *CPB* Cardiopulmonary bypass

*Significant effect modifiers

##### ICU LOS

**Fig. 4:**
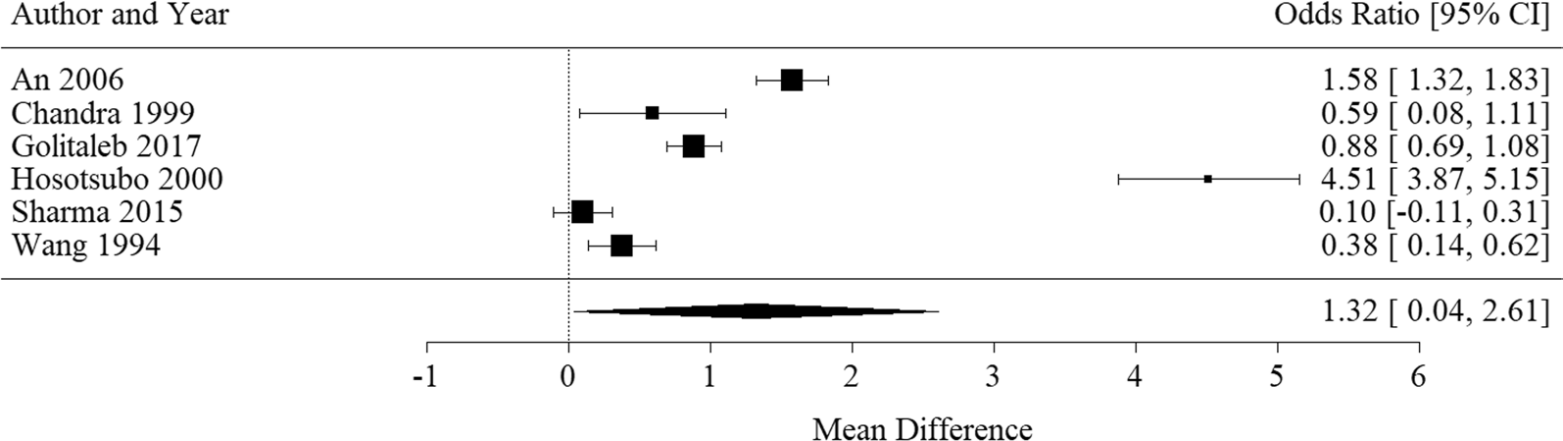
Forest plot for PH predicting ICU LOS

Six studies inclusive of 1974 patients reported ICU LOS [23, 26, 27, 31, 33, 34]. PH was associated with longer ICU LOS, albeit with marked heterogeneity (Mean difference 1.32 [95% CI 0.04, 2.6], I^2^ = 99.26%).) **(**see Fig. 4).

##### Hospital LOS

**Fig. 5:**
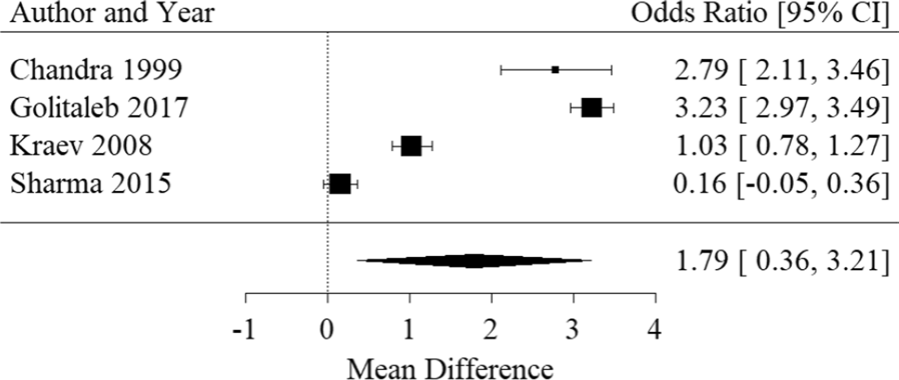
Forest plot for PH predicting hospital LOS

Four studies inclusive of 1979 patients reported hospital LOS [26, 28, 31, 34]. PH was associated with a longer hospital LOS, albeit with marked heterogeneity (Mean difference 1.79 [95% CI 0.36, 3.21], I ^2^= 99.03%) (see Fig. 5).

#### Qualitative analysis

##### Ventilation time

Two studies involving a total of 862 patients reported the relationship between PH and duration of mechanical ventilation [23, 31]. Both studies reported longer duration of mechanical ventilation in patients with PH; (25.3 ± 13.3 h vs 16.5 ± 9.2 h, *p* < 0.05) [23] and (23.92 ± 45.93 h vs 15.55 ± 34.32 h, *p* = 0.0001) [31].

##### Long term mortality

Diab et al. [25] reported 5-year mortality in 285 patients following surgery for infective endocarditis. Five-year survival was lowest in patients with preoperative liver dysfunction (20.1%) compared to 37.1% in patients with PH and 57% in patients with normal pre-operative liver function and no PH.

Kraev et al. [28] reported 2 year mortality in 826 patients following CPB. Patients were categorised into tertiles according to post-operative plasma bilirubin: group 1 (normal bilirubin levels), group 2 (1.4–2.8 mg/dL) and group 3 (> 2.8 mg/dL). Mortality at 24 months was 3.7% in patients with normal post-operative bilirubin and 16.7% in the upper tertile of plasma bilirubin (*p* < 0.001).

##### Phenotype of hyperbilirubinemia

PH incidence is higher in patients undergoing valvular surgeries compared to CABG only, and this finding is pronounced when multiple valves are operated on [23, 24, 26, 30, 31, 33, 34]. Most of the included studies differentiated between conjugated and unconjugated PH. Some attribute the observed PH as being predominantly conjugated bilirubin [24, 30], while others point to unconjugated bilirubin [33, 34]. The remaining studies suggest a mixed picture.

### Mortality rates

All studies except for two studies reported on the mortality rates observed in PH group vs non-PH group (see Additional file 4). The mean mortality rate in the PH group was 13.08% ± 9.35. The mean mortality rate in the non-PH group was 2.21% ± 2.38.

### Publication bias

**Fig. 6:**
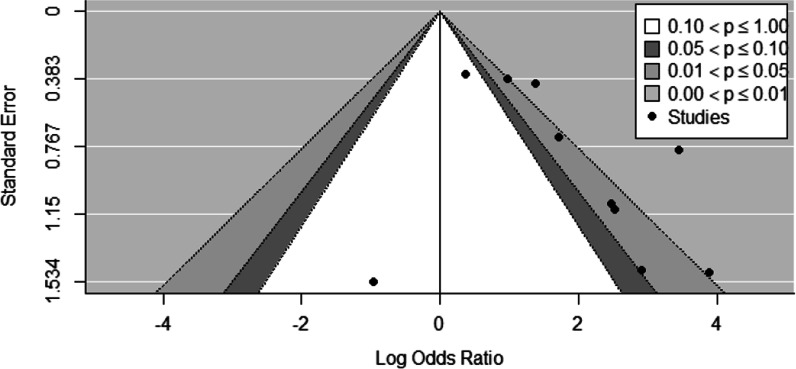
Contour Enhanced Funnel Plot

Publication bias was detected with the fixed-effects meta-regression model (*p* = 0.0152), but not the mixed-effects regression model for funnel asymmetry (*p* = 0.752) or the classical Egger test (*p* = 0.248). Visual inspection of asymmetry however shows slight asymmetry **(**see Additional file 5). Contour-enhanced funnel plot indicates suppression of studies reporting non-significant findings (see Fig. 6).

## Discussion

In this systematic review and meta-analysis, we found PH to be a promising prognostic biomarker for increased mortality and morbidity in cardiac surgery patients. The key finding of this study is that PH increases the odds of in-hospital mortality by sevenfold, especially in patients demonstrating persistent or late PH (POD > 7). The observed mortality rates were comparable to the figures reported by Australian and New Zealand Society of Cardiac and Thoracic Surgeons' Cardiac Surgery (ANZCTS) and the Society of Thoracic Surgeons (STS) [35, 36]. PH is also associated with longer ICU and hospital lengths of stay. Other covariates such as study year, age, bilirubin threshold, bilirubin monitoring period, CPB time, gender, proportion of CABG and proportion with existing pre-operative liver disease were not identified as significant modifiers. The overall methodological quality was poor due to high risk of bias and between-study heterogeneity was considerable, these factors may limit the generalisability of our findings, therefore further needed research will likely change our understanding of PH.

The meta-regression identified that the prognostic value of PH for in-hospital mortality increases with aortic cross-clamp time and number of blood units transfused. Longer cross-clamp times during cardiac surgery expose the patient to greater risks of low cardiac output, hypoxia and hypothermia which exacerbate hepatic injury [25, 26, 28, 30]. It is interesting to note that although total cross-clamp time was found to be a significant covariate in predicting in-hospital mortality, CPB time was not. Some studies provide support for this by demonstrating no significant difference in CPB time when comparing those that developed PH and those who did not [33]. Bilirubin accumulation from RBC hemolysis following perioperative blood transfusion can also increase risk of PH. Although this is reflected in our meta-regression, our observed transfusion requirements are marginally higher than what is indicated by current literature [5]. The inclusion of further studies with well-reported covariates are needed to increase the generalisability of our findings.

Several studies demonstrated preoperative liver disease to be a strong risk factor for PH, mortality, and morbidity [23–25, 27, 30, 31, 33, 34]. Our meta-regression did not detect a significant relationship between preoperative liver disease and the pooled odds ratio for in-hospital mortality. This may be due to the variability amongst authors in defining preoperative liver disease. Some defined preoperative liver disease using total bilirubin, others used aminotransferase derangements, and some used Model for End-Stage Liver Disease (MELD) scores. Similarly, there was variability amongst authors in how they obtained and defined the threshold value for hyperbilirubinemia. Consequently, we were unable to detect an effect-modifier relationship between hyperbilirubinemia threshold and the pooled odds ratio.

Qualitative analysis showed that patients with early PH characteristically demonstrate elevation of unconjugated bilirubin—most likely the result of the transient physiological insult by CPB, anaesthetics and blood transfusions [5]. These patients generally improve within 3 days[37]. The phenotype for patients with persistent or late PH (POD > 7) was predominantly conjugated. These patients were more likely to have long term hepatic dysfunction and multiple-organ failure due to systemic hypoperfusion, leading to increased mortality and morbidity [5, 10, 30]. The debate around the prognostic value of conjugated hyperbilirubinemia alone presents an interesting opportunity for future research. Only two studies reported on long term mortality, both suggesting PH is associated with poor long-term outcomes. More longitudinal studies are required to further investigate cause of death and morbidity in the long-term setting.

## Limitations

Insufficient reporting of relevant data and inconsistencies in the data reported prevented the inclusion of all relevant studies. This is compounded by the possible publication bias. Therefore, the predictive value of PH may be overstated and external validity to current practice maybe limited.

Secondly, there is a high level of residual heterogeneity due to insufficient reporting of patient and study level covariates. This in turn reduced both the precision of the pooled statistics and our ability to reliably perform sub-group analyses.

Only two studies performed multivariable analyses to adjust for potential confounders [25, 28]. Consequently, most studies were classified as having high risk of bias.

The range of PH in our included studies was 9% to 51%, with 3 of the most recent studies reporting PH rates of 25% [25, 26, 31]. Although these rates are consistent with epidemiological literature [5], more research is needed to interpret the variable incidence rates of PH. The relatively high incidence could be explained by the observation that while a portion of patients with biochemical PH will have clinical manifestations of hyperbilirubinemia, some will be restricted to an isolated (and clinically occult) biochemical event.

The low rate of observed deaths in the included studies reduced the confidence of our summary estimates for in-hospital mortality, ICU LOS and hospital LOS. Therefore, larger cohort studies with greater statistical power are needed to improve the confidence and precision of summary estimates.

## Implications for future research and practice

Qualitative analysis reveals CPB to be strong risk factor for PH and mortality, yet its complete effect on the human body remains to be understood. Therefore, additional CPB models should be developed to create safer and artificial circulation models.

The most widely ordered and investigated prognostic cardiac biomarkers are C-reactive protein (CRP), troponin, B-type natriuretic peptide (BNP), and N-terminal pro-BNP (Nt-pro-BNP) [38–40]. However, an array of new proteins, adhesion molecules and cytokines are also being investigated as potential prognostic biomarkers [41]. To our knowledge, this is the first systematic review and meta-analysis to synthesize the available evidence on the prognostic value of PH in cardiac surgery and to highlight the significance of early vs late PH peaks. The addition of PH to the list of newer prognostic haematological indices may aid in creating reliable predictive models for estimating mortality and morbidity in post-operative cardiac patients. Future research should focus on phenotyping PH as conjugated vs unconjugated and early vs delayed to determine which phenotypic profile confers the least and most favourable prognosis.

From a surgical standpoint, intra-operative considerations to prevent PH include aiming for reduced cross-clamp times and decreasing blood transfusion requirements. Patients with pre-existing heart failure or liver dysfunction require meticulous peri-operative planning and management [30]. Cardiac surgery is not recommended in patients with class C Child–Pugh cirrhosis [42]. Continual plasma bilirubin monitoring is paramount and persistent PH should prompt further investigations. Management of PH is mainly supportive with the main aim being the prevention of progression to hepatic failure, multi-organ failure and sepsis.

## Conclusion

PH is a promising prognostication tool predictive of in-hospital mortality. The timing of PH peaks may help differentiate between patients with transient hyperbilirubinemia, warranting longer ICU stay, from those with hepatic dysfunction, warranting longer hospital stay. Persistent PH should alarm the clinician of impending hepatic failure. Further high-quality studies that consistently report on patient level and study level co-variates are needed to reduce statistical heterogeneity and improve precision of summary estimates.

## Supplementary Information

**Additional file 1**. Search strategy (OVID Medline).

**Additional file 2**. Table of summary characteristics.

**Additional file 3**. Table of PROBAST assessment of Bias.

**Additional file 4**. Table of Mortality Rates.

**Additional file 5**. Funnel Plot for estimation of publication bias.

## Data Availability

Generated data that is not already included as supplementary material are available from the corresponding author, DR, upon reasonable request.

## Abbreviations

PH: Post-operative hyperbilirubinemia
ICU: Intensive care unit
CPB: Cardiopulmonary bypass
PRISMA: Preferred reporting items for systematic reviews and meta-analysis
PROBAST: The prediction model risk of bias assessment tool
CABG: Coronary artery bypass grafting
LD: Liver dysfunction
LOS: Length of stay
CB: Conjugated bilirubin
UCB: Unconjugated bilirubin
PICO: Population intervention comparator outcome
