# Supplementary material for: The prognostic significance of postoperative hyperbilirubinemia in cardiac surgery: systematic review and meta-analysis
Source: J Cardiothorac Surg. 2022 May 26;17:129. doi: 10.1186/s13019-022-01870-2 (PMC9137213; doi:10.1186/s13019-022-01870-2)
Supplement: Supplementary file 1 — Additional file 1. Search strategy (OVID Medline). [file 13019_2022_1870_MOESM1_ESM.docx]

Search Strategy (OVID Medline)

--------------------------------------------------------------------------------

1 Cardiac Surgical Procedures.mp. or exp Cardiac Surgical Procedures/ (220339)

2 Cardiopulmonary Bypass/ or Heart Bypass Left/ (23844)

3 ((cardiac or heart or coronary or pericardi*) adj4 (surger* or surgical or procedure* or operat*)).mp. (145666)

4 (valve adj4 (surger* or surgical or procedure* or operat*)).mp. (22822)

5 ((coronary adj4 bypass) or CABG).mp. (73023)

6 ((thoracic or cardiovascular or coronary or myocardial or pericardial or vascular or aorta or aorto*or valve or ventricular reconstructive or off pump or bypass) adj3 (surg* or operat*)).tw. (101069)

7 ((heart or aortocoronary or coronary or cardiopulmonary) adj5 (bypass or by pass)).tw. (81021)

8 ((valve* or arter*) adj5 (implant* or graft* or replac* or correct*)).tw. (106644)

9 (myocardial revasculari* or cavopulmonary or CABG).tw. (25292)

10 (angioplast* or (coronary adj3 balloon*) or cardiomyoplast*).tw. (46500)

11 (mitral valve replacement* or aortic valve repair* or mitral valve repair*).tw. (12766)

12 (maze surgery or arrhythmia surgery).tw. (224)

13 heart transplant.tw. (10161)

14 (ventricular adj5 (surg* or restor* or reconstruct*)).tw. (8500)

15 (heart failure surgery or hypertrophic cardiomyopathy surgery).tw. (42)

16 Aorta Thoracic/su (9104)

17 exp Cardiovascular Diseases/su (323973)

18 or/1-17 (624333)

19 Bilirubin.mp. [mp=title, abstract, original title, name of substance word, subject heading word, floating sub-heading word, keyword heading word, organism supplementary concept word, protocol supplementary concept word, rare disease supplementary concept word, unique identifier, synonyms] (49167)

20 Hyperbilirubinemia.mp. [mp=title, abstract, original title, name of substance word, subject heading word, floating sub-heading word, keyword heading word, organism supplementary concept word, protocol supplementary concept word, rare disease supplementary concept word, unique identifier, synonyms] (11725)

21 Hyperbilirubinemia.mp. [mp=title, abstract, original title, name of substance word, subject heading word, floating sub-heading word, keyword heading word, organism supplementary concept word, protocol supplementary concept word, rare disease supplementary concept word, unique identifier, synonyms] (11725)

22 conjugated hyperbilirubinemia.mp. [mp=title, abstract, original title, name of substance word, subject heading word, floating sub-heading word, keyword heading word, organism supplementary concept word, protocol supplementary concept word, rare disease supplementary concept word, unique identifier, synonyms] (379)

23 (severe adj hyperbilirubinemia).mp. [mp=title, abstract, original title, name of substance word, subject heading word, floating sub-heading word, keyword heading word, organism supplementary concept word, protocol supplementary concept word, rare disease supplementary concept word, unique identifier, synonyms] (450)

24 postoperative hyperbilirubinemia.mp. [mp=title, abstract, original title, name of substance word, subject heading word, floating sub-heading word, keyword heading word, organism supplementary concept word, protocol supplementary concept word, rare disease supplementary concept word, unique identifier, synonyms] (73)

25 Liver failure.mp. [mp=title, abstract, original title, name of substance word, subject heading word, floating sub-heading word, keyword heading word, organism supplementary concept word, protocol supplementary concept word, rare disease supplementary concept word, unique identifier, synonyms] (27387)

26 Liver Function Tests.mp. [mp=title, abstract, original title, name of substance word, subject heading word, floating sub-heading word, keyword heading word, organism supplementary concept word, protocol supplementary concept word, rare disease supplementary concept word, unique identifier, synonyms] (36186)

27 jaundice.mp. [mp=title, abstract, original title, name of substance word, subject heading word, floating sub-heading word, keyword heading word, organism supplementary concept word, protocol supplementary concept word, rare disease supplementary concept word, unique identifier, synonyms] (44542)

28 Icterus.mp. [mp=title, abstract, original title, name of substance word, subject heading word, floating sub-heading word, keyword heading word, organism supplementary concept word, protocol supplementary concept word, rare disease supplementary concept word, unique identifier, synonyms] (2989)

29 Hepatic Dysfunction.mp. [mp=title, abstract, original title, name of substance word, subject heading word, floating sub-heading word, keyword heading word, organism supplementary concept word, protocol supplementary concept word, rare disease supplementary concept word, unique identifier, synonyms] (4766)

30 (Serum adj bilirubin).mp. [mp=title, abstract, original title, name of substance word, subject heading word, floating sub-heading word, keyword heading word, organism supplementary concept word, protocol supplementary concept word, rare disease supplementary concept word, unique identifier, synonyms] (6829)

31 (blood adj Bilirubin).mp. [mp=title, abstract, original title, name of substance word, subject heading word, floating sub-heading word, keyword heading word, organism supplementary concept word, protocol supplementary concept word, rare disease supplementary concept word, unique identifier, synonyms] (264)

32 (bilirubin adj2 concentration).mp. [mp=title, abstract, original title, name of substance word, subject heading word, floating sub-heading word, keyword heading word, organism supplementary concept word, protocol supplementary concept word, rare disease supplementary concept word, unique identifier, synonyms] (1790)

33 (bilirubin adj testing).mp. [mp=title, abstract, original title, name of substance word, subject heading word, floating sub-heading word, keyword heading word, organism supplementary concept word, protocol supplementary concept word, rare disease supplementary concept word, unique identifier, synonyms] (34)

34 bilirubin toxicity.mp. [mp=title, abstract, original title, name of substance word, subject heading word, floating sub-heading word, keyword heading word, organism supplementary concept word, protocol supplementary concept word, rare disease supplementary concept word, unique identifier, synonyms] (190)

35 19 or 20 or 21 or 22 or 23 or 24 or 25 or 26 or 27 or 28 or 29 or 30 or 31 or 32 or 33 or 34 (147117)

36 Risk factors.mp. [mp=title, abstract, original title, name of substance word, subject heading word, floating sub-heading word, keyword heading word, organism supplementary concept word, protocol supplementary concept word, rare disease supplementary concept word, unique identifier, synonyms] (1082412)

37 Long-term outcomes.mp. [mp=title, abstract, original title, name of substance word, subject heading word, floating sub-heading word, keyword heading word, organism supplementary concept word, protocol supplementary concept word, rare disease supplementary concept word, unique identifier, synonyms] (33303)

38 mortality.mp. [mp=title, abstract, original title, name of substance word, subject heading word, floating sub-heading word, keyword heading word, organism supplementary concept word, protocol supplementary concept word, rare disease supplementary concept word, unique identifier, synonyms] (1215849)

39 morbidity.mp. [mp=title, abstract, original title, name of substance word, subject heading word, floating sub-heading word, keyword heading word, organism supplementary concept word, protocol supplementary concept word, rare disease supplementary concept word, unique identifier, synonyms] (417382)

40 (in-hospital adj mortality).mp. [mp=title, abstract, original title, name of substance word, subject heading word, floating sub-heading word, keyword heading word, organism supplementary concept word, protocol supplementary concept word, rare disease supplementary concept word, unique identifier, synonyms] (25202)

41 Acute kidney injury.mp. [mp=title, abstract, original title, name of substance word, subject heading word, floating sub-heading word, keyword heading word, organism supplementary concept word, protocol supplementary concept word, rare disease supplementary concept word, unique identifier, synonyms] (59291)

42 AKI.mp. [mp=title, abstract, original title, name of substance word, subject heading word, floating sub-heading word, keyword heading word, organism supplementary concept word, protocol supplementary concept word, rare disease supplementary concept word, unique identifier, synonyms] (14824)

43 (blood adj1 transfusion).mp. [mp=title, abstract, original title, name of substance word, subject heading word, floating sub-heading word, keyword heading word, organism supplementary concept word, protocol supplementary concept word, rare disease supplementary concept word, unique identifier, synonyms] (83078)

44 atrial fibrillation.mp. [mp=title, abstract, original title, name of substance word, subject heading word, floating sub-heading word, keyword heading word, organism supplementary concept word, protocol supplementary concept word, rare disease supplementary concept word, unique identifier, synonyms] (89539)

45 post-operative atrial fibrillation.mp. [mp=title, abstract, original title, name of substance word, subject heading word, floating sub-heading word, keyword heading word, organism supplementary concept word, protocol supplementary concept word, rare disease supplementary concept word, unique identifier, synonyms] (228)

46 afib.mp. [mp=title, abstract, original title, name of substance word, subject heading word, floating sub-heading word, keyword heading word, organism supplementary concept word, protocol supplementary concept word, rare disease supplementary concept word, unique identifier, synonyms] (362)

47 length of stay.mp. [mp=title, abstract, original title, name of substance word, subject heading word, floating sub-heading word, keyword heading word, organism supplementary concept word, protocol supplementary concept word, rare disease supplementary concept word, unique identifier, synonyms] (125412)

48 LOS.mp. [mp=title, abstract, original title, name of substance word, subject heading word, floating sub-heading word, keyword heading word, organism supplementary concept word, protocol supplementary concept word, rare disease supplementary concept word, unique identifier, synonyms] (83312)

49 low output syndrome.mp. [mp=title, abstract, original title, name of substance word, subject heading word, floating sub-heading word, keyword heading word, organism supplementary concept word, protocol supplementary concept word, rare disease supplementary concept word, unique identifier, synonyms] (580)

50 36 or 37 or 38 or 39 or 40 or 41 or 42 or 43 or 44 or 45 or 46 or 47 or 48 or 49 (2556191)

51 18 and 35 and 50 (1020)

******
