# Supplementary material for: The prognostic significance of postoperative hyperbilirubinemia in cardiac surgery: systematic review and meta-analysis
Source: J Cardiothorac Surg. 2022 May 26;17:129. doi: 10.1186/s13019-022-01870-2 (PMC9137213; doi:10.1186/s13019-022-01870-2)
Supplement: Supplementary file 3 — Additional file 3. Table of PROBAST assessment of Bias. [file 13019_2022_1870_MOESM3_ESM.docx]

|  | OVERALL ASSESSMENT | Participants |  |  | Predictors |  |  |  | Outcome |  |  |  |  |  |  | Analysis |  |  |  |  |  | not relevant | not relevant | not relevant |  |
| --- | --- | --- | --- | --- | --- | --- | --- | --- | --- | --- | --- | --- | --- | --- | --- | --- | --- | --- | --- | --- | --- | --- | --- | --- | --- |
| Study ID |  | 1.1 Were Approriate Data Sources Used | 1.2 Were All Inclusions/Exclusions Appropriate | TOTAL | 2.1 Were Predictors defined and assessed in similar way for all participants | 2.2 Were predictor assessments made without knowledge of outcome assessments | 2.3 Are all predictors available at the time the model is to be used | TOTAL | 3.1 Was the outcome determined appropriately | 3.2 Was a prespecified out standard outcome definition used | 3.3 Were predictors excluded from outcome definition | 3.4 Was the outcome defined and determined in a similar way for all participants | 3.5 Was the outcome determined without knowledge of predictor information | 3.6 Was the time interval between predictor and outcome appropriate | TOTAL | 4.1 Was there a reasonable number of participants with the outcome | 4.2 Were continous and categorical predictors handled appropriately | 4.3 Were all enrolled participants included | 4.4 Was missing data handled appropriately | 4.5 Was selection of predictors based on univariable analysis avoided | 4.6 Were complexities in data accounted for (multivariate analysis) | 4.7 Were relevant model performance measures handled appropriately (calibration/discrimination) | 4.8 Were model over and underfitting and optimisation of model performance accounted for | 4.9 Do predictors and their assigned weights in the final model correspond to the results in multivariable analysis | TOTAL |
| Collins 1983 | **High** | Y | PY - criteria too simple | **Low** | Y | Y | Y | **Low** | Y | Y | Y | Y | PY | PN | **Low** | N | Y | Y | No info | N | N | No Info | No info | No info | **High** |
| Wang 1994 | **High** | Y | PY - study includes preop hyperbilirubin bilirubin patients | **Low** | Y | Y | Y | **Low** | Y | Y | Y | Y | PY | PY | **Low** | N | Y | Y | No info | N | N | No Info | No info | No info | **High** |
| Chandra 1999 | **High** | Y | PY - criteria too simple | **Low** | Y | Y | Y | **Low** | Y | Y | Y | Y | PY | PY | **Low** | N | Y | Y | No info | Y | N | No Info | No info | No info | **High** |
| Hosotsubo 2000 | **High** | Y | Y | **Low** | Y | PN | Y | **Low** | Y | Y | Y | Y | N | PY | **Low** | N | Y | Y | No info | Y | N | No Info | No info | No info | **HIgh** |
| An 2006 | **High** | Y | Y | **Low** | Y | Y | Y | **Low** | Y | Y | Y | Y | PY | PY | **Low** | Y | Y | Y | No info | Y | N | No Info | No info | No info | **High** |
| Leacche 2006 | **Low** | Y | Y | **Low** | Y | PN | Y | **Low** | Y | Y | Y | Y | N | PN | **High** | N | Y | N | No info | Y | Y | PN | PY | PY | **Low** |
| Kraev 2008 | **High** | Y | Y | **Low** | Y | PN | Y | **Low** | Y | Y | Y | Y | N | PY | **Low** | Y | Y | Y | No info | N | Y | No Info | No info | No info | **High** |
| Vidal 2009 | **Low** | Y | Y | **Low** | Y | Y | Y | **Low** | Y | Y | Y | Y | PY | Y | **Low** | N | Y | Y | No info | Y | Y | No Info | No info | No info | **Low** |
| Nishi 2012 | **High** | Y | Y | **Low** | Y | Y | Y | **Low** | Y | Y | Y | Y | PY | PY | **Low** | Y | Y | Y | No info | N | N | No Info | No info | No info | **High** |
| Sharma 2015 | **High** | Y | Y | **Low** | Y | Y | Y | **Low** | Y | Y | Y | Y | PY | Y | **Low** | N | Y | Y | No info | Y | N | No Info | No info | No info | **High** |
| Diab 2017 | **High** | Y | Y | **Low** | Y | PN | Y | **Low** | Y | Y | Y | Y | N | PY | **Low** | Y | Y | Y | No info | N | N | No Info | No info | No info | **High** |
| Golitaleb 2017 | **High** | Y | Y | **Low** | Y | Y | Y | **Low** | Y | Y | Y | Y | PY | Y | **Low** | Y | Y | Y | No info | N | N | No Info | No info | No info | **High** |

Table of PROBAST Scores
