# Supplementary material for: The prognostic significance of postoperative hyperbilirubinemia in cardiac surgery: systematic review and meta-analysis
Source: J Cardiothorac Surg. 2022 May 26;17:129. doi: 10.1186/s13019-022-01870-2 (PMC9137213; doi:10.1186/s13019-022-01870-2)
Supplement: Supplementary file 4 — Additional file 4. Table of Mortality Rates. [file 13019_2022_1870_MOESM4_ESM.docx]

**Online Supplement_4_Mortality Rates**

**Mortality rates**

| **Study ID** | **Mortality in PH group (n)** | **Mortality in PH group (%)** | **Mortality in non-PH group, n (%)** | **Mortality in non-PH group (%)** | **OR** | **Lower CI** | **Upper CI** |
| --- | --- | --- | --- | --- | --- | --- | --- |
| **Collins 1983** | 12/49 | (25%) | 2/197 | (1%) | 31.00 | 6.79 | 147.16 |
| **Wang 1994** | 6/106 | (5.6%) | 1/196 | (0.5%) | 11.09 | 1.32 | 93.38 |
| **Chandra 1999** | 0/20 | (0%) | 3/57 | (5.3%) | 0.44 | 0.02 | 8.93 |
| **Hosotsubo 2000** | 8/68 | (11.7%) | 0/65 | (0%) | 18.40 | 1.04 | 325.76 |
| **An 2006** | 4/97 | (4.1%) | 1/289 | (0.3%) | 12.39 | 1.37 | 112.21 |
| **Leacche 2006** | NR |  | NR |  | 5.60 | 2.1 | 15.00 |
| **Kraev 2008** | 18/72 | (25%) | 13/754 | (1.7%) | 19.00 | 8.84 | 40.83 |
| **Vidal 2009** | NR |  | NR |  | 1.44 | 1.12 | 1.84 |
| **Nishi 2012** | 13/63 | (20.6%) | 3/271 | (1.1%) | 22.97 | 6.31 | 83.54 |
| **Sharma 2015** | 14/119 | (11.8%) | 17/357 | (4.8%) | 2.23 | 1.01 | 4.92 |
| **Diab 2017** | 13/54 | (24.1%) | 17/231 | (7.4%) | 2.67 | 1.27 | 5.59 |
| **Golitaleb 2017** | 5/150 | (3%) | 0/450 | (0%) | 27.02 | 1.48 | 491.95 |
| **Overall** |  | 13.08% |  | 2.21% |  |  |  |

The mean mortality rate in the PH group is 13.08% (9.35)

The mean mortality rate in the non PH group is 2.21% (2.38).
