# Supplementary figures and images for: The prognostic significance of postoperative hyperbilirubinemia in cardiac surgery: systematic review and meta-analysis
Source: J Cardiothorac Surg. 2022 May 26;17:129. doi: 10.1186/s13019-022-01870-2 (PMC9137213; doi:10.1186/s13019-022-01870-2)

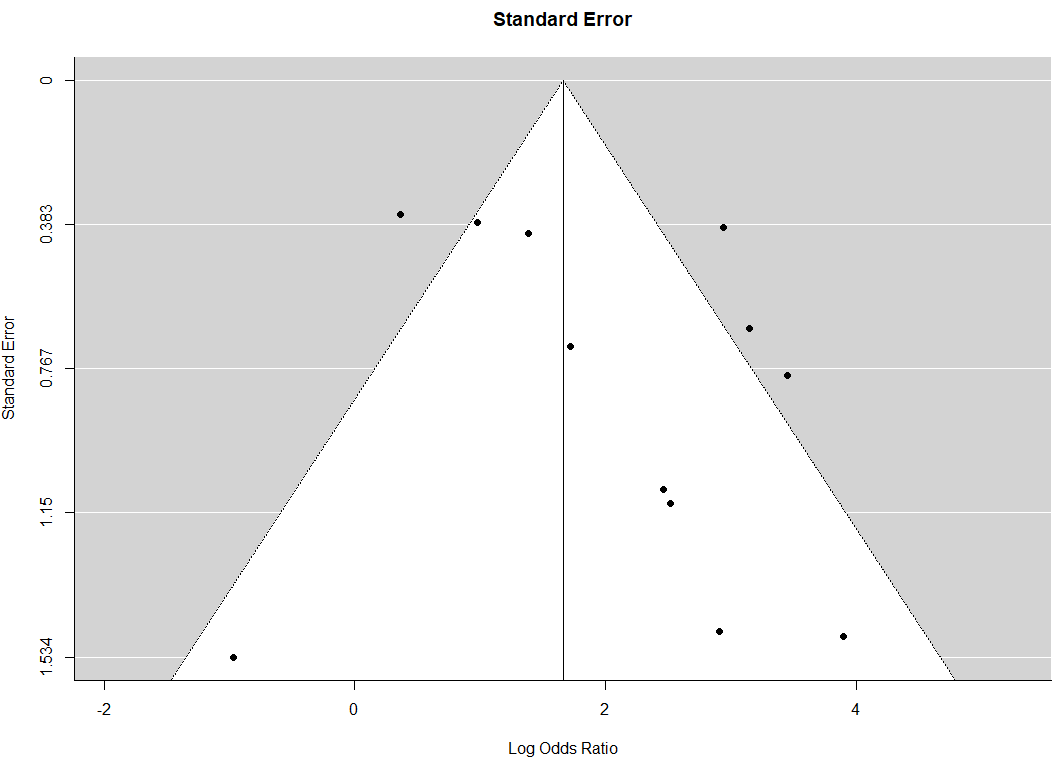


Funnel plot for estimation of publication bias

Supplement: Supplementary file 5 — Additional file 5. Funnel Plot for estimation of publication bias. [file 13019_2022_1870_MOESM5_ESM.docx]
